# An airborne actinobacteria *Nocardiopsis alba* isolated from bioaerosol of a mushroom compost facility

**DOI:** 10.1007/s10453-014-9336-4

**Published:** 2014-04-17

**Authors:** Mariola Paściak, Krzysztof Pawlik, Andrzej Gamian, Bogumiła Szponar, Justyna Skóra, Beata Gutarowska

**Affiliations:** 1 Laboratory of Medical Microbiology, Ludwik Hirszfeld Institute of Immunology and Experimental Therapy, Polish Academy of Sciences, Rudolfa Weigla 12, 53-114 Wrocław, Poland; 2 Laboratory of Molecular Biology of Microorganisms, Ludwik Hirszfeld Institute of Immunology and Experimental Therapy, Polish Academy of Sciences, Rudolfa Weigla 12, 53-114 Wrocław, Poland; 3 Institute of Fermentation Technology and Microbiology, Technical University of Łódź, Wólczańska 171/173, 90-924 Lodz, Poland

**Keywords:** Actinobacteria, Compost, Bioaerosol, Airborne microorganisms, *Nocardiopsis alba*, Occupational exposure, Indoor microbial exposure

## Abstract

*Actinobacteria* are widely distributed in many environments and represent the most important trigger to the occupant respiratory health. Health complaints, including hypersensitivity pneumonitis of the workers, were recorded in a mushroom compost facility (MCF). The studies on the airborne bacteria were carried out to find a possible microbiological source of these symptoms. Culture analysis of compost bioaerosols collected in different location of the MCF was performed. An assessment of the indoor microbial exposure revealed bacterial flora of bioaerosol in the mushroom compost facility represented by *Bacillus*, *Geobacillus*, *Micrococcus*, *Pseudomonas*, *Staphylococcus* spp., and actinobacterial strain with white aerial mycelium. The thermotolerant actinobacterial strain of the same morphology was repeatedly isolated from many locations in MCF: air, compost sample, and solid surface in production hall. On the base of complex morphological, chemotaxonomic, and phylogenetic characteristics, the isolate has been classified as *Nocardiopsis alba*. Dominant position of *N. alba* in microbial environment of the mushroom compost facility may represent an indicator microorganism in compost bioaerosol. The bioavailability of *N. alba* in mushroom compost facility creates potential risk for the health of workers, and the protection of respiratory tract and/or skin is strongly recommended.

## Introduction

A harmful occupational and environmental microbial exposure has been recognized in agriculture for decades. Members of the class *Actinobacteria* are predominant among airborne environmental microorganisms and represent an important trigger to human respiratory health. Airborne spores of *Saccharopolyspora*, *Streptomyces*, and *Thermoactinomyces* genera (*Firmicutes*) are responsible for hypersensitivity pneumonitis known as farmer’s or mushroom worker’s lung (Zacharisen and Fink 2011; Xu et al. 2002; Moore et al. 2004) and other severe health effects (Kagen et al. 1981; Lacey and Dutkiewicz 1994).

Spore-forming actinobacteria of the *Nocardiopsis* genus are opportunistic pathogens rarely encountered in clinical practice. Nevertheless, *N. dassonvillei* is known as etiologic agent of mycetoma (Sindhuphak et al. 1985), skin lesions, alveolitis (Bernatchez and Lebreux 1991), and pulmonary infections (Mordarska et al. 1998). Diagnostic tests based on biochemical reactions applied in clinical microbiology often do not include actinobacteria; therefore, chemotaxonomic analyses are recommended. Chemotaxonomic markers of the genus *Nocardiopsis* are comprised of a characteristic fatty acid and polar lipids profile with phosphatidylcholine (Lechevalier et al. 1977) and two major glycolipids (Mordarska et al. 1998). The other chemical markers of *Nocardiopsis* are peptidoglycan component *meso*-2,6-diaminopimelic acid (*meso*-DAP) and the major menachinone (MK-10). There are no characteristic sugars in whole-cell hydrolysates.

Here, we describe an actinobacterial strain isolated from the air of a mushroom compost production facility (MCF) in central Poland. The occupational health service had been informed about a number of respiratory disorders among the workers directly involved in a production, as well as a co-owner of the facility employed in the office. The office worker developed hypersensitivity pneumonitis, a chronic respiratory disorder. To find a possible microbiological cause of these afflictions, the airborne bacteria were isolated and cultivated. *Actinobacteria* were found in many locations of the mushroom compost facility. The predominate thermotolerant aerial mycelium-producing bacterial strain was selected for identification. The complex morphological, chemotaxonomic, and phylogenetic characteristics were performed for the identification of the airborne actinobacterial strain.

## Materials and methods

### Description of test locations

The composting plant producing compost for mushroom growing was evaluated for environmental bacteriological contamination. The following locations were selected: production and sale halls (number of samples *N* = 9); laboratory and offices in factory (*N* = 7); entrance (*N* = 2); the factory owners’ house, located in a distance of 100 m from factory (*N* = 9); house entrance (*N* = 2); the owners’ cars (*N* = 2). Outdoor air samples (ca 2 km from the mushroom compost facility) were also collected (*N* = 5).

### Isolation and cultivation of the bacteria

The samples of air, compost, and from surfaces in the MCF were collected during winter months (November–February 2009). Air samples were collected using a MAS-100 Eco Air Sampler (Merck Millipore). Fifty or hundred liters of air was aspirated and propelled onto a 90-mm Petri dish with either TSA medium (Tryptic Soy Agar, Merck, Germany) or YPG medium (yeast extract–peptone glucose medium; yeast extract 10 g L^−1^; peptone 20 g L^−1^, glucose 20 g L^−1^, pH 7.2). Solid media were prepared with the addition of nystatin (0.2 %). Samples were incubated at 37 ± 2 and 55 ± 2 °C for 48 and 72 h. Bacterial colonies were counted and reported as cfu m^−3^ (colony-forming units per cubic meter).

In parallel, samples were also taken from surfaces such as walls and equipment of the production hall, to confirm the presence of bacteria from air sampling. The surface samples were collected using Envirocheck^®^ Contact Plates (Merck, Germany) with TSA medium. The air and surface samples were taken in 3–5 repetitions, depending on the premise volume. The presence of bacteria was confirmed in compost samples: 1 g of compost was weighed and mixed with 99 ml of sterile saline, and then a series of dilutions were prepared. The samples of surfaces and compost were incubated on TSA medium at 37 ± 2 and 55 ± 2 °C for 48 and 72 h.

The bacteria were identified by macroscopic and microscopic features observation (color, texture, size, dyes), Gram staining, catalase, oxidase test (Microbiologie Bactident Oxydase, Merck, Germany), and using API^®^ tests (bioMérieux, France): API^®^50 CH (performance of carbohydrate metabolism tests), API^®^Staph (identification of staphylococci and micrococci), API^®^20E (identification of *Enterobacteriaceae* and other non-fastidious Gram-negative bacteria), API^®^20NE (identification of Gram-negative non-*Enterobacteriaceae*).

On the basis of the results, one actinobacterial strain with aerial mycelium, which was dominating in each location (air, surface, and compost samples), was chosen and subjected to further taxonomic identification. An isolated airborne actinobacterial strain was deposited in the Polish Collection of Microorganisms as PCM 2702, preserved in 20 % glycerol solution, and frozen at −70 °C.

### Reference bacterial strains

The reference type strains from microbiological collections were used during study: *Nocardiopsis alba* PCM 2496 (DSM 43377), *N. alborubida* PCM 2490 (DSM 40465), *N. antarcticus* PCM 2489 (JCM 6843), *N. prasina* PCM 2493 (JCM 3336), and *N. dassonvillei* PCM 2492 (JCM 7437).

### Morphology and pigmentation analyses

Colony morphology and pigmentation were observed on the following media: nutrient agar (NA), glucose–yeast extract–malt extract (GYM agar), medium 79 (peptone 10 g L^−1^, yeast extract 2 g L^−1^, casein hydrolysate 2 g L^−1^, NaCl 6 g L^−1^, glucose 20 g L^−1^, pH 7.5), oatmeal agar (ISP medium 3), inorganic salts-starch agar (ISP medium 4), potato dextrose agar (PDA), tryptone-soya agar (TSA), tryptone yeast extract (TYE), yeast extract–malt extract (MYA), yeast extract–peptone glucose (YPG), and cultivated for 7 days at 37 °C (Shirling and Gottlieb 1966).

### Physiological characteristics and biochemical tests

The optimal growth condition for an actinobacterial isolate was checked at 22, 28, 37, 45, 50, and 55 °C on GYM agar, medium 79, nutrient, and blood agar for 7 days.

The tolerance of different NaCl concentrations (0, 3, 5, 10, 15, 20, 25, 30 % w/v) was determined on GYM agar incubated for 7 days at 37 °C.

A full panel of physiological tests: hydrolysis of starch, gelatin, casein, esculin, adenine, hypoxanthine, xanthine, tyrosine; utylization of d-xylose, d-glucose, l-arabinose, l-rhamnose, d-raffinose, d-mannitol, fructose, cellulose, sucrose, and *meso*-inositol; decomposition of urea was performed (Yassin et al. 1997).

#### Antibiotic susceptibility

Minimal inhibitory concentration (MIC) of antibiotics was tested against the airborne actinobacteria strain PCM 2702 and *N. alba* PCM 2496 and *N. prasina* PCM 2493 using discs of amoxicillin, erythromycin, gentamicin, tetracycline, and vancomycin, in concentration of 5, 10, and 30 µg on GYM agar incubated for 5 days at 37 °C. The amikacin and oflaxacin were studied in concentration of 30 and 5 µg, respectively.

### Chemotaxonomic studies

An airborne actinobacterial isolate PCM 2702 biomass was obtained by cultivation on medium 79 in the orbitally shaken flasks (for the aeration of bacterial suspension), during 48 h at 37 °C. Bacteria were killed in Koch apparatus (1 h, 100 °C), centrifuged at 11,000*g* (Sigma), and washed twice by PBS and water. One part of biomass was freeze-dried; the remaining part was stored at −70 °C prior to chemotaxonomic analyses.

Chemotaxonomic identification of the airborne actinobacterial isolate was based on the analyses of the whole-cell sugars, DAP isomer in peptidoglycan, the cellular fatty acids, and the polar lipid profile in the dry and wet biomass (Paściak et al. 2007).

Whole-cell sugars and DAP isomers in whole-cell hydrolysates were determined according to Schaal (1985).

Fatty acid analysis of the whole-cell dry biomass was performed after methanolysis in 2 M methanolic HCl at 80 °C (Paściak et al. 2007). Fatty acid methyl esters (FAMES) were analyzed by gas–liquid chromatography coupled with mass spectrometry (Focus GC with ion trap ITQ 700, Thermo) using helium as the carrier gas and a temperature program on Rxi^®^-5 ms/Restek column (30 m × 0.25 mm ID) of 150–270 °C, 12 °C/min.

Crude lipid for polar lipid analysis was extracted from dry cell mass by chloroform–methanol (2:1, v/v) at 37 °C according to Bligh–Dyer method (Kates 1986). Polar lipids: phospholipids and glycolipids were analyzed by TLC in chloroform–methanol–water solvent system and visualized by Dittmer and Lester, and orcinol reagent, respectively (Mordarska and Paściak 1994).

Mycolic acids were extracted and analyzed by thin-layer chromatography (TLC) according to Embley and Wait (1994).

### Genotypic identification

Chromosomal DNA was prepared using a DNA extraction kit dedicated to *Streptomyces* (A&A Biotechnology, Poland). The 16S rRNA gene was enzymatically amplified using the universal oligonucleotide primers 16S start (AGAGTTTGATCMTGGCTCAG) and 16S stop (AAGGAGGTGWTCCARCC) as in Chun and Goodfellow (1995). PCR products were purified and sequenced directly (Genomed Joint-Stock Company, Poland); in parallel, the obtained PCR products were cloned into pGEM^®^-T Easy vector systems and sequenced.

The identification of phylogenetic neighbors was initially carried out by the BLASTN (Altschul et al. 1997) programs against the database of type strains with validly published prokaryotic names (Kim et al. 2012). The 30 sequences with the highest scores were then selected for the calculation of pairwise sequence similarity using global alignment algorithm (Myers and Miller 1988), which was implemented at the EzTaxon server (http://eztaxon-e.ezbiocloud.net/) (Kim et al. 2012).

Phylogenetic trees were reconstructed using MEGA software version 5.05 (Tamura et al. 2011) for the neighbor-joining method with Kimura two-parameter model and minimum-evolution and maximum-parsimony methods (Takahashi and Nei 2000) and PHYML (Guindon et al. 2005) for the maximum-likelihood method. The topologies of the trees were evaluated by bootstrap analysis (Felsenstein 1985) based on 1,000 resamplings (neighbor-joining, minimum-evolution and maximum-parsimony, and maximum-likelihood). 16S rRNA gene sequence similarity was calculated using the EzTaxon server.

## Results

### Isolation and cultivation of the airborne actinobacteria

Airborne bacteria were detected in all study locations; actinobacteria were encountered near the compost packing machine and other locations in the mushroom compost facility, as well as in the office/laboratory building and the owner’s house and cars (Table 1). The highest number of actinobacteria was observed in mushroom compost production hall (2.6 × 10^3^ cfu m^−3^); high values were found also in laboratory and office buildings (Table 1).

**Table 1:** Airborne bacteria collected in the mushroom compost facility and its vicinity

Location	Number of samples	Total bacteria (cfu m^−3^)^a^	Aerial mycelium actinobacteria (cfu m^−3^)^a^
Mushroom compost facility	9	M: 4.5 × 10^4^ SD: 1.6 × 10^4^	M: 2.6 × 10^3^ SD: 9.3 × 10^2^
Laboratory/office building entrance	2	M: 5.3 × 10^4^ SD: 7.1 × 10^1^	M: 2.4 × 10^3^ SD: 5.7 × 10^2^
Laboratory/office building	7	M: 9.3 × 10^3^ SD: 1.4 × 10^4^	M: 7.5 × 10^2^ SD: 1.0 × 10^3^
Owners’ house entrance	2	M: 4.8 × 10^3^ SD: 1.4 × 10^3^	M: 7.4 × 10^1^ SD: 8.3 × 10^1^
Owners’ house	9	M: 7.2 × 10^2^ SD: 4.2 × 10^2^	M: 1.5 × 10^2^ SD: 1.3 × 10^2^
Owners’ cars	2	M: 8.0 × 10^2^ SD: 9.9 × 10^2^	M: 1.0 × 10^1^ SD: 1.4 × 10^1^
Outdoor air in the vicinity of the MCF	5	M: 6.0 × 10^1^ SD: 4.9 × 10^1^	M: 0.0 SD: 0.0

^a^ *M* mean, *SD* standard deviation

The identification results of airborne bacteria in the production hall of the MCF, microorganisms present on solid surfaces, and in compost are shown in Table 2. Only one actinobacterial strain (PCM 2702) was found in bioaerosol, in the compost sample, and on the solid surfaces; four bacterial species (*Bacillus* sp., *Geobacillus thermoglucosidasius*, *Methylobacterium mesophilicum*, and *Micrococcus lylae*) were present in bioaerosols and in compost. The majority of the strains were isolated from bioaerosol only (Table 2).

**Table 2:** Bacteria detected in air, solid surface, and compost in mushroom compost facility (production hall)

Strain	Places of isolation		
	Air	Solid surface^a^	Compost
*Bacillus cereus*	−	+	−
*Bacillus lentus*	+	−	−
*Bacillus licheniformis*	−	−	+
*Bacillus* sp.	+	−	+
*Brevibacillus* sp.	−	−	+
*Brevundimonas vesicularis*	−	−	+
*Geobacillus* sp.	+	−	−
*Geobacillus thermoglucosidasius*	+	−	+
*Kocuria varians*	+	−	−
*Methylobacterium mesophilicum*	+	−	+
*Leuconostoc mesenteroides*	+	+	−
*Micrococcus lylae*	+	−	+
*Micrococcus* sp.	+	−	−
*Pseudomonas mendocina*	−	+	−
*Pseudomonas stutzeri*	+	−	−
*Staphylococcus lentu*s	+	−	−
*Staphylococcus sciuri*	+	−	−
*Actinobacterial strain/Nocardiopsis* sp.	+	+	+

^a^Walls and equipment of production hall

Based on these results, the predominant actinobacterial strain in MCF was selected for identification, deposited in Polish Collection of Microorganisms as PCM 2702 and subjected to further cultivation prior to establish its taxonomic position.

### Morphology of the actinobacterial isolate

**Fig. 1:**
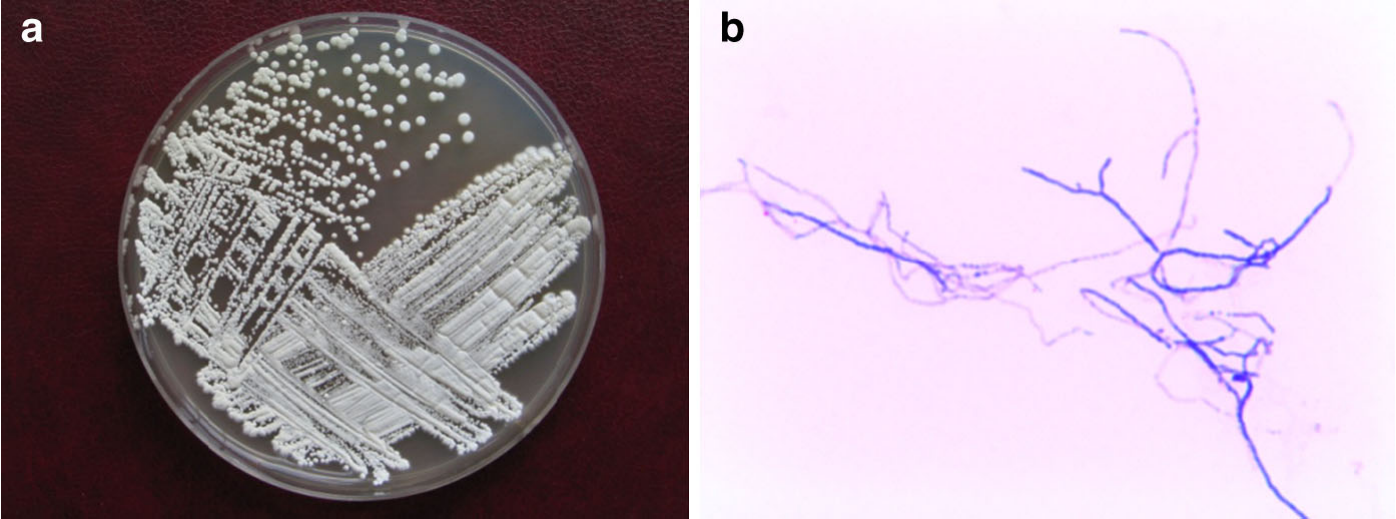
Airborne actinobacteria PCM 2702 isolated from the mushroom compost facility. **a** Nutrient agar, 30 °C, 72 h; **b** Gram staining, light microscopy ×1,200

The airborne actinobacterial strain PCM 2702 was found to be a white aerial mycelium-producing bacterial strain, Gram-positive, aerobic, and non-motile organism with long and branched filaments (Fig. 1). This strain grew well aerobically on nutrient agar, GYM agar, ISP medium 3, TSA, TYE, MYA, and YPG agar. On these media, the substrate mycelium was cream colored and the aerial mycelium was white. The colonies were of irregular edges. No diffusible pigment was produced (Fig. 1).

### Physiological characteristics

The strain PCM 2702 grew up to 45 °C degree with the optimum at 28–37 °C; growth at 22 °C was not observed.

The strain grew in 10 % NaCl (w/v) similarly to the collection strain *N. alba* PCM 2496. Airborne actinobacterial strain PCM 2702 was catalase-positive, produced nitrate reductase and H_2_S. Physiological characteristics for type strains of *N. alba* and *N. prasina* and airborne strain studied are summarized in Table 3. The airborne isolate PCM 2702 differed from *N. alba* and *N. prasina* collection strains in rhamnose utilization as a sole carbon source for growth.

**Table 3:** Differential phenotypic characteristics of airborne strain PCM 2702, *N. alba* PCM 2496, and *N. prasina* PCM 2493

Characteristics	Airborne isolate PCM 2702	*N. alba* PCM 2496	*N. prasina* PCM 2493
Hydrolysis of			
Starch	+	+	+
Gelatin	+	+	+
Casein	+	+	+
Esculin	−	−	+
Adenine	+	+	+
Hypoxanthine	+	+	+
Xanthine	+	+	+
Tyrosine	+	+	+
Utilization of the following compounds as carbon sources			
d-Xylose	−	−	−
d-Glucose	+	+	−
l-Arabinose	−	−	−
l-Rhamnose	+	−	−
d-Raffinose	−	−	−
d-Mannitol	+	+	−
Fructose	+	+	−
Cellulose	+	+	+
Sucrose	+	+	+
*Meso*-inositol	−	−	−
Decomposition of urea	+	+	−

### Antibiotic susceptibility

The MIC was measured for an airborne isolate PCM 2702 and *N. alba* PCM 2496*, N. prasina* PCM 2493: amoxicillin ≤10 µg, amikacin ≤30 µg, erythromycin ≤10 µg, gentamicin ≤10 µg, tetracycline ≤10 µg, vancomycin ≤5 µg. Ofloxacin revealed MIC of ≤5 µg for *N. alba* and airborne isolate PCM 2702 but *N. prasina* was not susceptible for this antibiotic in concentration studied.

### Chemotaxonomy

Chemotaxonomic studies revealed that strain PCM 2702 had a chemical profile consistent with *Nocardiopsis* genus. In whole-cell hydrolysates of the strain PCM 2702, *meso*-DAP was present and no characteristic sugars were detected (III/C cell wall chemotype according to Lechevalier and Lechevalier 1970). The fatty acid profile consisted of branched C14:0, C15:0, C16:0, C17:0, C18:0, and 10-methyl branched C18:0 (TBS) fatty acids (Table 4). Hydroxy fatty acids, including mycolic acids, were not detected. The fatty acid profile of airborne strain PCM 2702 was similar to *N. alba* PCM 2496 and differed from *N. prasina* PCM 2493 in terms of TBS content.

**Table 4:** Fatty acid analysis of airborne strain PCM 2702, *N. alba* PCM 2496, and *N. prasina* PCM 2493;  % of total fatty acids

	Airborne isolate PCM 2702	*N. alba* PCM 2496	*N. prasina* PCM 2493
*iso*-14:0	0.58	0.68	2.23
*anteis*o-15:0	1.77	1.73	7.58
*iso*-16:0	47.54	41.26	37.97
16:0	1.78	4.13	6.46
*iso*-17:0	1.1	1.08	0
*anteiso*-17:0	7.88	10.4	15.84
17:0	0	0	6.67
br-18:0	3.54	3.59	0
*iso/anteiso*-18:0	10.85	8.9	2.24
18:1	0.73	1.29	4.09
18:0	4.3	6.33	16.02
TBS	19.94	20.61	0.89

The major phospholipids of an airborne isolate PCM 2702 were phosphatidylcholine, phosphatidyl methylethanolamine, diphosphatidylglycerol, phosphatidylglycerol (phospholipid type III, Lechevalier et al. 1977).

**Fig. 2:**
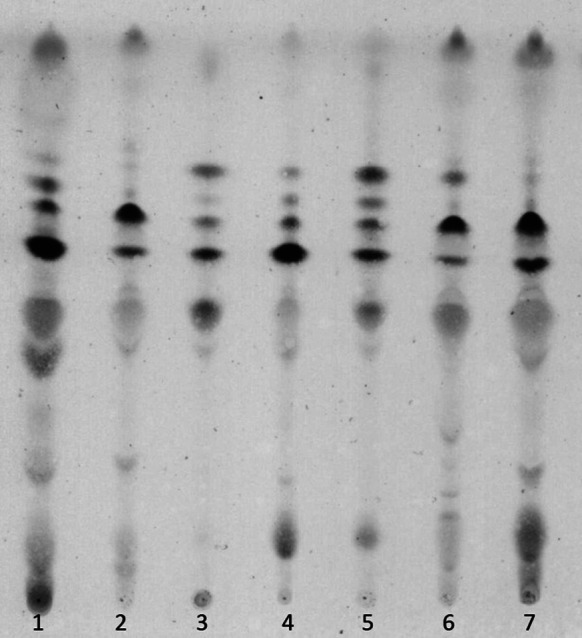
TLC of glycolipids from *Nocardiopsis* spp.: 1. *N. dassonvillei*, 2. *N. alborubida*, 3. Airborne actinobacteria isolate PCM 2702, 4. *N. alba*, 5. Airborne actinobacteria isolate PCM 2702, 6. *N. prasina*, and 7. *N. antarcticus*. Solvent system: chloroform–methanol–water (65:25:4 v/v/v). Detection: orcinol reagent

Among polar lipids, four major glycolipids were detected; two of them, G1 and G2, characteristic for *Nocardiopsis* genus (Mordarska et al. 1998), were reported (Fig. 2).

### 16S rRNA sequence analysis

**Fig. 3:**
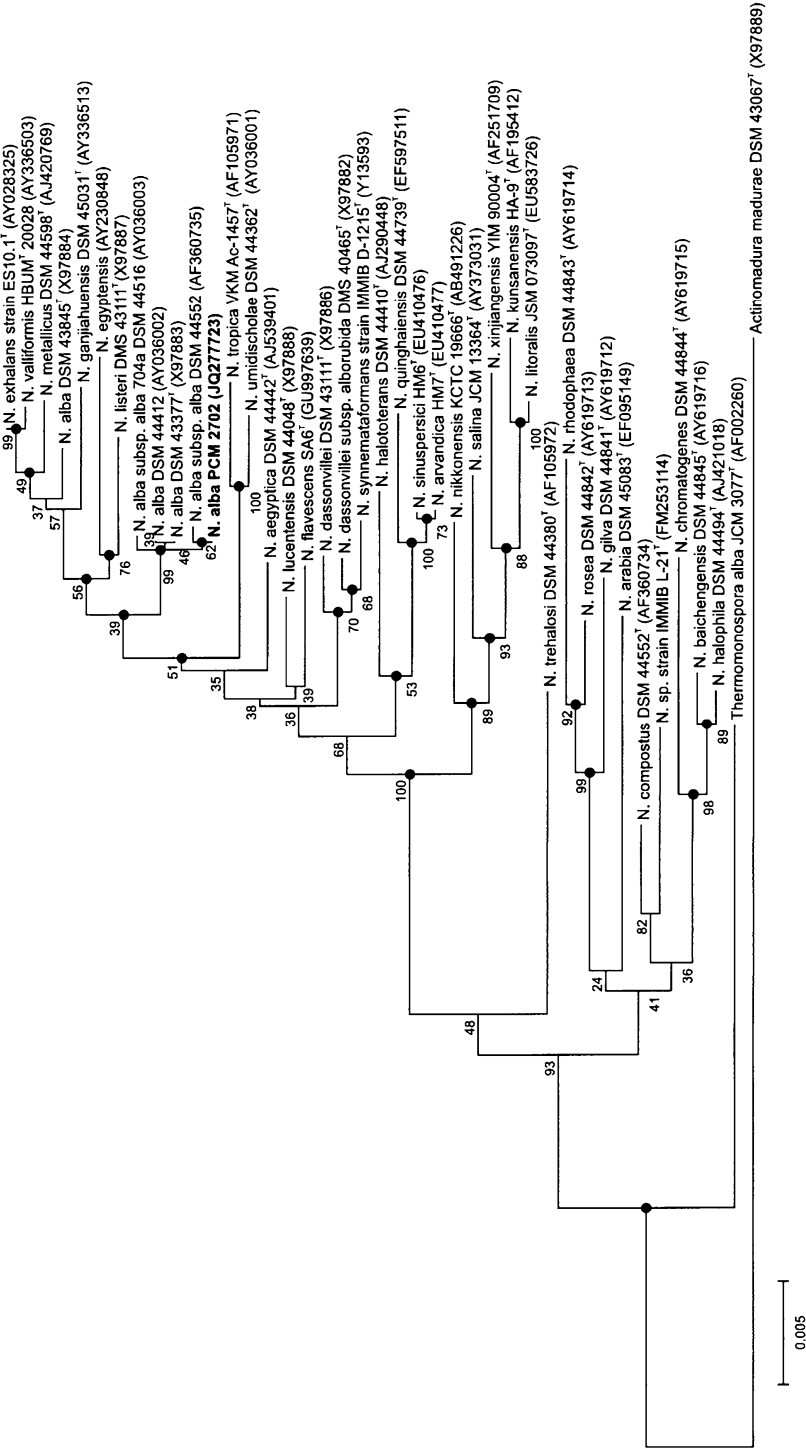
Neighbor-joining tree showing the position of strain PCM 2702 in the genus *Nocardiopsis*

To establish the phylogenetic position of the strain PCM 2702, its 16S rDNA gene sequence was determined in the study (1,525 bp) (GenBank accession no JQ277723). Sequence similarity calculations indicated that the closest relatives of an airborne isolate PCM 2702 were *N. alba* DSM 43377^T^ (Grund and Kroppenstedt 1990): 99.932 %; *N. exhalans* ES10.1^T^ (Peltola et al. 2001): 99.105 %; *N. prasina* DSM 43845^T^ (Yassin et al. 1997): 98.973 %; *N. valliformis* HBUM 20028^T^ (Yang et al. 2008): 98.953 %; *N. lucentensis* DSM 44048^T^ (Yassin et al. 1993): 98.904 %. Phylogenetic tree was constructed, showing the nearest phylogenetic relatives of the strain PCM 2702 in Fig. 3.

Strain PCM 2702 revealed 99.93 % 16S rRNA gene sequence similarity to *N. alba* DSM 43377^T^. In conclusion, according to genotypic and phenotypic data, an airborne actinobacterial isolate PCM 2702 was proved to represent a new strain of *N. alba* species.

## Discussion

Bacterial flora of bioaerosol in the mushroom compost facility studied here is represented by *Bacillus*, *Geobacillus*, *Micrococcus*, *Pseudomonas*, and *Staphylococcus* genera. This is rather typical and similar also to other types of compost (e.g., green waste composting plants). In the air samples collected in different compost facilities were identified strains of *Bacillus humi*, *B. niabensis*, *B. coagulans*, *Geobacillus thermodenitrificans*, *Pseudomonas* sp. (Karadag et al. 2013), and *Leuconostoc pseudomesenteroides* (Bru-Adan et al. 2009).

Actinobacteria in bioaerosols of composting plants constitute common flora, especially thermophilic and thermotolerant representatives have been identified: *Saccharomonospora*, *Thermobifida*, *Saccharopolyspora*, *Mechercharimyces* (Karadag et al. 2013; Le Goff et al. 2011; Schäfer et al. 2013).

Currently, genus *Nocardiopsis* harbors twelve validly described species and one subspecies (Kroppenstedt and Evtushenko 2006). Of these, the majority were isolated from soils and one (*N. compostus*) from the bioaerosol in a composting facility (Kämpfer et al. 2002).

The strain PCM 2702 isolated in the mushroom compost facility reported here was a dominant microorganism in bioaerosol. The strain was thermotolerant (as in nearly all *Nocardiopsis* species) and revealed the optimal growth temperature at 28 °C (Kroppenstedt and Evtushenko 2006).

Physiologic properties and chemotaxonomic studies revealed that the airborne isolate PCM 2702 had a chemical profile consistent with *Nocardiopsis* genus, i.e., cell wall chemotype III, the fatty acid profile, the phospholipid type, and characteristic glycolipids. Especially, the fatty acid and glycolipid patterns are significant taxonomic markers for *Nocardiopsis*. 16S rDNA gene sequence analysis revealed that the strain represents *N. alba* taxon.

Worth to note, *N. alba* strain was present in the production hall, the office, and the owner’s house, but never detected in outdoor air sampled in a distance of 1 km away from the MCF, suggesting a close relation to microbial environment of the mushroom compost facility.

The availability of large amount of spore-forming actinomycetes in occupational environments can pose potential health problems. The most prevalent form of hypersensitivity pneumonitis is caused mainly by *Saccharopolyspora rectivirgula*, *Thermoactinomyces vulgaris,* and *T. candidus* (McNeil and Brown 1994, Schäfer et al. 2013). Recently, a case of farmer’s lung was described, where positive reaction against *Aspergillus fumigatus*, *Aspergillus terreus,* and *N. alba* was recorded by immunodetection (Imai et al. 2004). So it cannot be ruled out that *N. alba* caused hypersensitivity pneumonitis in the occupants of the studied buildings.

The role of *Nocardiopsis dassonvillei* in respiratory tract disorders was reported previously in a study of aerobic actinomycetes involved in human infections in Nigeria, were among 41 patients with bronchopulmonary disorders, *N. dassonvillei* was detected in two patients (Gugnani et al. 1998), and our group found *N. dassonvillei* to be the sole etiological agent of severe pulmonary infection (Mordarska et al. 1998). The identification by glycolipid markers was confirmed by DNA/DNA homology studies.

The actinobacterial strain *N. alba* PCM 2702 isolated in the compost producing facility was strongly dominated in the environment and clearly represented an occupational exposure for the employees. This could be supported by the reporting of one case of hypersensitivity pneumonitis in an office worker and, although not clinically confirmed, chronic headaches and weakness of the others workers and the owner’s family members living in a house in a close distance to the facility.

The bioavailability of *N. alba* in mushroom compost facility creates potential risk for health for workers, and the protection of respiratory tract and/or skin is strongly recommended. Studies on pathogenicity and allergic properties of *N. alba* will be continued.

## Abbreviations

PCM: Polish Collection of Microorganisms
DSM: Deutsche Sammlung von Mikroorganismen
JCM: Japan Collection of Microorganisms
MCF: Mushroom compost facility
YPG: Yeast extract–peptone glucose medium
cfu: Colony-forming units
TLC: Thin-layer chromatography
